# Laboratory early warning score versus clinical early warning score as a predictor of imminent cardiac arrest

**DOI:** 10.1186/cc13271

**Published:** 2014-03-17

**Authors:** M Keil, S Hutchinson, T Leary

**Affiliations:** 1 Norfolk and Norwich University Hospital NHS Foundation Trust, Norwich, UK

## Introduction

NCEPOD reported in 2012 that 75% of patients had warning signs for cardiac arrest present prior to their arrest [1]. NICE recommends a vital sign-based early warning score (EWS) to identify patients at risk of deterioration or death [2]. In our trust, audit has shown that only 20 to 35% of patients trigger the clinical EWS prior to cardiac arrest. Jarvis and colleagues proposed that an EWS based on common laboratory findings can predict patient mortality [3]. The aim of this study, as part of a wider review of cardiac arrests in our hospital, was to determine whether the laboratory early warning score (LEWS) might be of use identifying patients at risk of cardiac arrest in our trust.

## Methods

Retrospective data collected identified cardiac arrest calls that lead to CPR or defibrillation over 6 months. The LEWS was calculated according to the formula devised by Jarvis and colleagues [3]. LEWS ≥4 for males and ≥5 for females was taken as being a 'trigger' as suggested by Jarvis and colleagues [3].

## Results

Eighty-nine cardiac arrest calls lead to CPR and/or defibrillation in this time period. Of these, 65 patients had had blood tests within the last 24 hours. Median LEWS was 6 for females and 7 for males (range 1 to 12). Most patients (77%) had a LEWS trigger (≥4 for females and ≥5 for males).

## Conclusion

The collected data suggest that more patients at risk of cardiac arrest in our hospital might be identified using a LEWS rather than a clinical EWS. It is evident that for a clinical EWS regular observations need to be taken and are subject to user error. Clinical EWS could also not be sensitive enough or there is a failure to implement it successfully. LEWS can be generated automatically when bloods are taken. The downside is that it relies on bloods being done. It is beyond the scope of this study to examine the sensitivity/specificity of LEWS or suggest that LEWS should replace EWS. We suggest that LEWS may compliment EWS by identifying a different group of patients. The ongoing data collection aims to correlate the clinical EWS for each patient directly with their LEWS to confirm the initial findings.
